# Multimodal Assessment of Breast Cancer-Related Lymphedema Using Bioimpedance Analysis, Ultrasound Tissue Thickness, and Shear Wave Elastography

**DOI:** 10.3390/diagnostics16162594

**Published:** 2026-08-16

**Authors:** Mi-Hyeon Bang, Ha-Lim Song, Geunyeol Jo, Hwan-Kwon Do

**Affiliations:** 1 Department of Physical Medicine and Rehabilitation, Haeundae Paik Hospital, Inje University College of Medicine, Busan 48108, Republic of Korea; h80862@paik.ac.kr (M.-H.B.); h80861@paik.ac.kr (H.-L.S.); hprm@paik.ac.kr (G.J.); 2 Department of Physical Medicine and Rehabilitation, Dongnam Institute of Radiological & Medical Sciences (DIRAMS), Busan 46033, Republic of Korea

**Keywords:** bioelectrical impedance analysis, breast cancer-related lymphedema, stage-specific, multimodal assessment, shear wave elastography, tissue thickness, ultrasound

## Abstract

**Background/Objectives:** Lymphedema is a chronic condition caused by impaired lymphatic function that is characterized by fluid accumulation with progression to tissue thickening and fibrosis. Although various noninvasive assessment methods have been proposed, studies that have integrated fluid, structural, and stiffness-related changes are limited. To address this gap, we conducted an integrated multimodal assessment combining multiple-frequency bioelectrical impedance analysis (MFBIA), ultrasound tissue thickness measurements, and shear wave elastography (SWE) to characterize stage-specific changes in breast cancer-related lymphedema. **Methods:** This prospective cross-sectional study included patients who had undergone breast cancer surgery. Participants were classified into the clinical and subclinical lymphedema groups based on MFBIA criteria. Dermal thickness, subcutaneous thickness, and tissue stiffness measurements in both upper limbs were obtained using SWE on the same day. MFBIA parameters including extracellular water ratio, 5 kHz impedance ratio, and phase angle were assessed. Ipsilateral and contralateral limbs were compared, and correlation analyses of variables were performed. **Results:** Dermal thickness and subcutaneous thickness in the ipsilateral limb were greater than those in the contralateral limb in the overall cohort and subclinical group. Increased tissue stiffness measured using SWE was observed only in the clinical lymphedema group. MFBIA parameters in the clinical group were higher than those in the subclinical group. According to the correlation analysis, MFBIA parameters were associated with tissue thickness and SWE parameters were not correlated with other variables. **Conclusions:** These findings support the clinical utility of a multimodal approach for the stage-specific evaluation of breast cancer-related lymphedema, thereby facilitating stage-based and individualized management.

## 1. Introduction

Lymphedema is a chronic condition characterized by the accumulation of protein-rich fluid in the interstitial space caused by impaired lymphatic transport [1,2,3]. Under normal physiological conditions, the lymphatic system maintains fluid homeostasis by removing interstitial fluid and macromolecules that leak from capillaries. However, structural or functional abnormalities of the lymphatic system lead to impaired lymphatic drainage, resulting in lymphostasis. This results in increased intralymphatic pressure, valvular insufficiency, and reduced lymph transport capacity, which ultimately cause the accumulation of fluid and protein in the skin and subcutaneous tissues [2]. The persistent accumulation of lymphatic fluid, proteins, and glycosaminoglycans induces activation of fibroblasts, keratinocytes, and adipocytes, thus leading to dermal thickening and progressive fibrosis of subcutaneous tissue [2,4].

Lymphedema is broadly classified into primary lymphedema, which is caused by congenital abnormalities of the lymphatic system, and secondary lymphedema, which develops following trauma, infection, surgery, or radiation therapy [1,2]. Lymphedema is estimated to affect approximately 140–250 million individuals worldwide, thus representing a significant global health burden [5]. Breast cancer-related lymphedema (BCRL), which is one of the most common and clinically important forms of lymphedema, occurs in approximately 16–21% of patients following breast cancer treatment [5].

The risk of lymphedema among patients who have undergone axillary lymph node dissection is approximately four-fold higher than that of patients who have undergone sentinel lymph node biopsy, and the majority of cases develop within the first year after treatment, although the risk persists for several years postoperatively [5,6,7]. Beyond limb swelling, lymphedema is associated with a wide range of physical symptoms, including pain, heaviness, and restricted range of motion, as well as a significant reduction in quality of life [8]. Therefore, an accurate and stage-specific assessment is essential for preventing disease progression and improving clinical outcomes. Prospective surveillance programs have been proposed to facilitate early detection and management of BCRL [7,9,10,11].

In clinical practice, the diagnosis and staging of lymphedema are primarily based on the patient history and physical examination findings. Key clinical features include limb swelling, pitting edema, a positive Stemmer sign, and progressive changes in tissue composition and skin characteristics [12]. These findings are incorporated in the staging system proposed by the International Society of Lymphology [13]. However, such clinical assessments lack sensitivity for detecting early-stage lymphedema because the diagnosis typically occurs only after the development of visible morphological changes, which often correspond to more advanced stages. Additionally, a subclinical stage exists in which lymphatic dysfunction is present without overt physical changes, thus making early detection particularly challenging [12].

To overcome these limitations, various quantitative and imaging-based assessment methods, including circumferential measurements, volumetric analysis, bioimpedance spectroscopy, lymphoscintigraphy, magnetic resonance imaging, and ultrasonography, have been introduced. However, each modality has inherent limitations. Circumferential and volumetric measurements primarily reflect global limb volume changes and are unable to capture subtle fluid shifts or early structural alterations in the skin and subcutaneous tissues [2,14]. Moreover, these methods are subject to operator dependency, limited sensitivity, and time-consuming procedures, thus restricting their utility as early diagnostic tools. Because each modality captures different aspects of the underlying pathophysiology, a single assessment method is often insufficient to comprehensively characterize the condition; therefore, interest in multimodal approaches that integrate complementary techniques for a more complete evaluation of lymphedema is increasing.

Imaging modalities such as lymphoscintigraphy and indocyanine green lymphography enable visualization of lymphatic flow and identification of lymphatic obstruction. However, these techniques primarily assess lymphatic fluid dynamics and provide limited information regarding tissue-level changes, such as fibrosis or structural remodeling within the skin and subcutaneous tissues [15,16]. Moreover, although ICG lymphography provides high-resolution visualization of lymphatic function, intense pain at the injection site related to the dye’s pH remains a significant hurdle in the patient experience, and the technique requires specialized near-infrared imaging equipment [17].

The multiple-frequency bioelectrical impedance analysis (MFBIA) is a noninvasive method that evaluates body fluid distribution by measuring the electrical impedance of tissue. It is particularly sensitive to changes in extracellular fluid, can detect abnormalities even before the clinical onset of lymphedema, and has high sensitivity for identifying subclinical disease [18,19,20].

Ultrasound is a noninvasive, readily available, and cost-effective imaging modality that allows detailed evaluations of soft tissue structures and subcutaneous changes in patients with BCRL. Previous studies have demonstrated that characteristic ultrasonographic findings in the ipsilateral limb include morphological alterations of the skin and subcutaneous tissues, such as increased thickness and changes in echogenicity. Fluid accumulation within the subcutaneous tissue can be visualized as hypoechoic areas on ultrasound imaging. As disease progresses, these areas may present with a characteristic “stone-paved” or cobblestone appearance reflecting excessive interstitial fluid accumulation. These features provide valuable information for assessing disease presence and progression in BCRL. However, although ultrasound enables quantitative measurements of skin thickness and subcutaneous thickness, its diagnostic performance may be limited by operator dependency and challenges in differentiating lymphedema from other causes of edema [21,22].

Ultrasound elastography has emerged as a complementary noninvasive technique that evaluates tissue mechanical properties beyond conventional morphological assessments. Ultrasound elastography includes two main techniques: strain elastography and shear wave elastography (SWE). Strain elastography is operator-dependent and relies on external compression, whereas SWE provides quantitative assessments of tissue stiffness by measuring the propagation speed of shear waves, which is directly proportional to tissue stiffness. SWE has been widely applied in liver, thyroid, and breast imaging and is increasingly used in musculoskeletal imaging for the noninvasive quantitively assessment of tissue stiffness [23,24]. In lymphedema, SWE has been proposed as a promising modality for assessing fibrotic changes and tissue rigidity [25,26,27,28]. However, elastography primarily reflects mechanical tissue properties and provides limited information regarding fluid dynamics within tissues, thus representing an inherent limitation of the technique.

Most previous studies have focused on individual assessment modalities; therefore, integrated analyses of how fluid accumulation, structural alterations, and mechanical changes interact throughout the progression of lymphedema are lacking. In particular, studies that have investigated the relationships between these parameters in the subclinical stage, prior to the development of overt lymphedema, are limited.

In this study, we performed an integrated evaluation of lymphedema using the MFBIA, ultrasound-based tissue thickness measurements, and SWE. We also compared patients with clinical and subclinical lymphedema to characterize stage-specific biomarker profiles and investigate the relationships among fluid, structural, and mechanical changes. This multimodal approach may facilitate a more comprehensive stage-specific evaluation of breast cancer-related lymphedema.

## 2. Materials and Methods

### 2.1. Participants

This prospective cross-sectional study was conducted at a single center between August and November 2023. Patients who visited the outpatient clinic of the Department of Rehabilitation Medicine were consecutively enrolled. Eligible participants were required to meet the following inclusion criteria: female patients with a history of unilateral breast cancer surgery; underwent surgery at least 3 months before study enrollment; and prior sentinel lymph node biopsy or axillary lymph node dissection. Exclusion criteria included the following: history of bilateral breast cancer surgery; comorbid conditions associated with upper extremity edema, such as heart failure, renal failure, infection, or thrombophlebitis; recent trauma to the upper limb; and pre-existing upper extremity edema prior to breast cancer surgery.

All participants provided written informed consent after receiving a detailed explanation of the study procedures. The study protocol was approved by the Institutional Review Board of Haeundae Paik Hospital.

Demographic and clinical data, including age, body mass index, comorbidities, breast cancer surgery side, lymph node surgery type, and history of chemotherapy and radiotherapy, were collected through medical record reviews and patient interviews. The limb on the side of surgery was defined as the ipsilateral limb, and the contralateral limb was used as the reference limb. All measurements were performed on the same day to ensure consistency.

Patients were classified into groups based on the MFBIA. Clinical lymphedema was defined using the interlimb MFBIA ratio thresholds of ≥1.139 when the dominant arm was ipsilateral and ≥1.066 when the nondominant arm was ipsilateral based on established clinical guidelines [20].

### 2.2. Bioelectrical Impedance Analysis

The MFBIA was performed for all participants using the InBody S10 device (InBody Co., Ltd., Seoul, Republic of Korea). Measurements were conducted with the participants in a standardized position according to the manufacturer’s guidelines. Segmental parameters of both upper limbs were obtained, including the extracellular water (ECW), impedance at 5 kHz, and phase angle.

The ECW/total body water ratio was calculated for each limb, and the interlimb ECW/total body water ratio (ipsilateral/contralateral) was used as an indicator of edema severity.

Impedance measured at a low frequency (5 kHz) reflects the electrical properties of extracellular fluid because cell membranes act as capacitors at low frequencies, thus restricting current flow to the extracellular compartment. Accordingly, the interlimb 5 kHz impedance ratio was used to assess extracellular fluid-related changes [20,29].

The phase angle, which was calculated based on resistance and reactance, reflects cellular membrane integrity and cellular function, and it is widely used as an indicator of cellular health and physiological status [30,31,32]. The interlimb phase angle ratio was used to evaluate relative differences in tissue conditions between limbs.

### 2.3. Ultrasound and Shear Wave Elastography

Ultrasound examinations were performed using the HS70A ultrasound system (Samsung Medison, Seoul, Republic of Korea) equipped with a linear-array transducer in S-shearwave mode. All measurements were conducted by a single experienced physiatrist under the supervision of a board-certified neuroradiologist.

**Figure 1:**
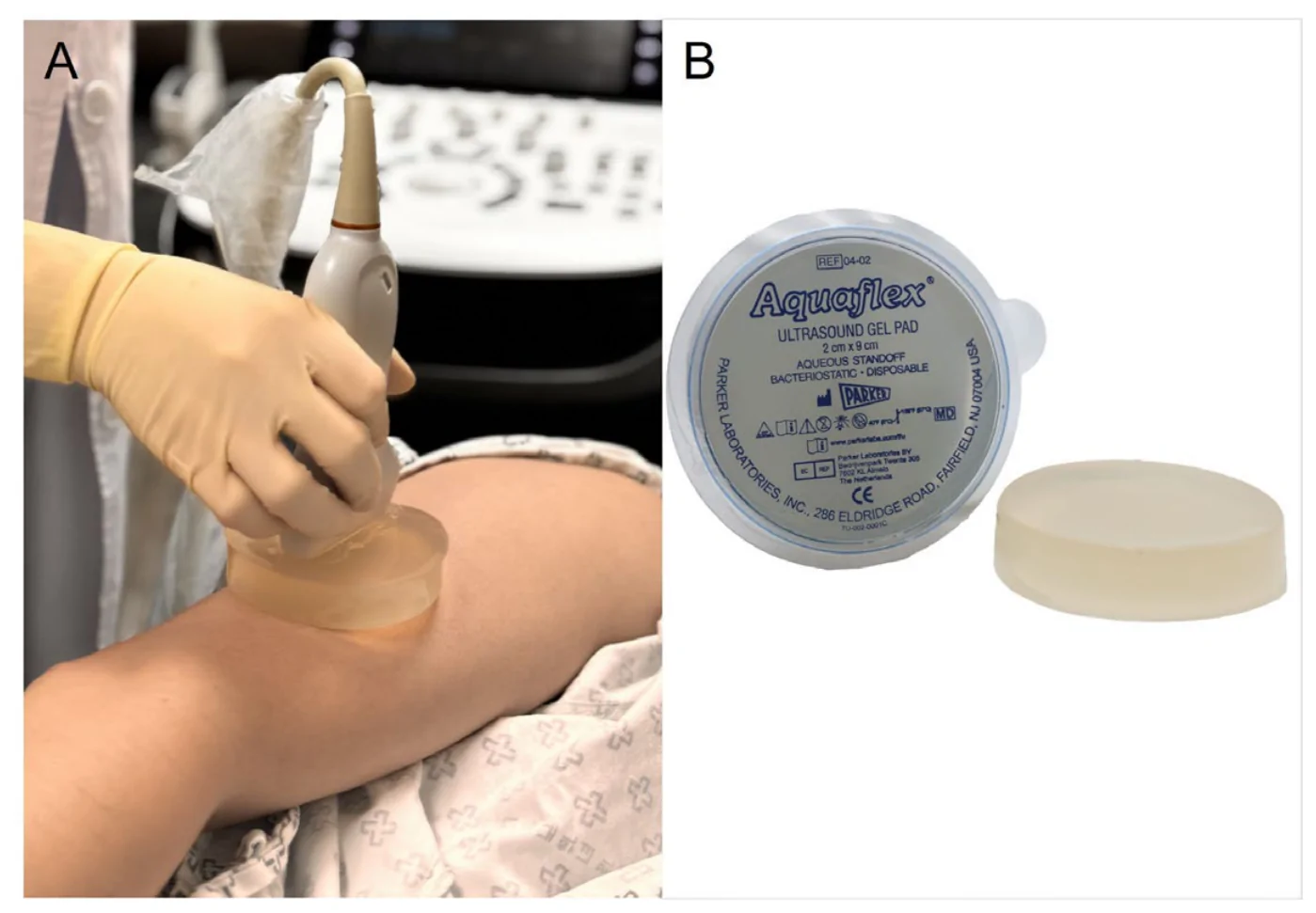
Ultrasound and shear wave elastography examination protocol. (**A**) Measurements were performed with the participant in the lateral decubitus position. The upper limb was assessed at sites located 10 cm proximal and 10 cm distal to the elbow crease. An Aquaflex ultrasound gel pad (Parker Laboratories) was used to minimize tissue compression caused by transducer pressure. (**B**) The Aquaflex ultrasound gel pad (Parker Laboratories) used during all ultrasound and shear wave elastography examinations.

Participants were positioned in the lateral decubitus position with the arm fully extended during the examination. Measurement sites were defined at 10 cm proximal (upper arm) and 10 cm distal (forearm) to the antecubital fold. The ultrasound probe was initially placed parallel to the antecubital fold to obtain transverse B-mode images. Subsequently, the probe was rotated to align with the longitudinal axis of the limb to acquire SWE images. An ultrasound gel pad (Aquaflex; Parker Laboratories, Fairfield, NJ, USA) was used to minimize probe compression artifacts and improve visualization of superficial tissues during ultrasound and SWE examinations (Figure 1) [33,34].

Dermal thickness and subcutaneous thickness were measured on B-mode images. Tissue stiffness was evaluated using shear wave velocity (SWV; m/s).

**Figure 2:**
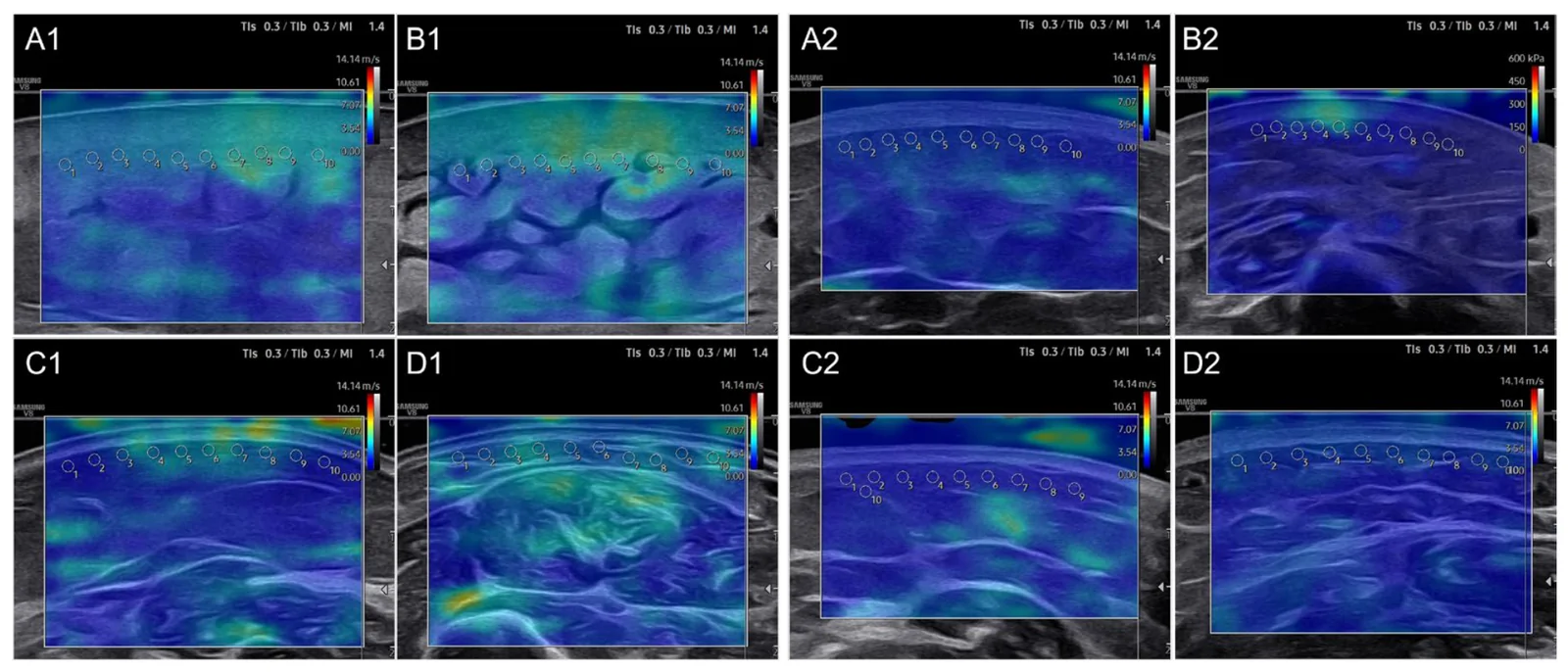
Representative shear wave elastography (SWE) images of patients with clinical and subclinical lymphedema. (**A1**–**D1**) SWE images of a representative patient with clinical lymphedema: ipsilateral upper arm (**A1**), ipsilateral forearm (**B1**), contralateral upper arm (**C1**), and contralateral forearm (**D1**). (**A2**–**D2**) Images of a representative patient with subclinical lymphedema: ipsilateral upper arm (**A2**), ipsilateral forearm (**B2**), contralateral upper arm (**C2**), and contralateral forearm (**D2**). Color-coded SWE maps illustrate tissue stiffness. Ten small circular regions of interest (ROIs), numbered 1–10 in each image, were placed within the subcutaneous tissue along the longitudinal axis of the limb for the quantitative stiffness assessment.

Because lymphedematous tissue exhibits heterogeneous characteristics, including fluid accumulation, adipose changes, and fibrosis, the use of a single region of interest (ROI) may result in sampling bias. To minimize this issue, ten circular ROIs (5 × 5 mm) were distributed within the subcutaneous tissue at each measurement site (Figure 2). Only ROIs with a Reliable Measurement Index (RMI) ≥ 0.5 were included in the analysis. Measurements with an RMI < 0.5 were immediately repeated during the examination and were not included in the final analysis.

For each measurement site, the median SWV value from the ten ROIs was used as the representative value to reduce the influence of outliers. Additionally, measurements were repeated three times at each site, and the mean of the median SWV values from the three repeated measurements was used for the final analysis. The examiner was blinded to the MFBIA findings and the surgical side throughout the ultrasound and SWE examinations.

### 2.4. Statistical Analysis

All statistical analyses were performed using SPSS software (version 25.0; IBM Corp., Armonk, NY, USA). Continuous variables were presented as the mean ± standard deviation. Normality of the data distribution was assessed using the Shapiro–Wilk test. Within-subject comparisons of the ipsilateral and contralateral limbs were performed using the Wilcoxon signed-rank test. Differences between the clinical and subclinical lymphedema groups were analyzed using the Mann–Whitney U test. Correlations between variables were assessed using Spearman’s correlation analysis. All tests were two-tailed, and *p* < 0.05 was considered statistically significant.

## 3. Results

### 3.1. Baseline Characteristics

A total of 53 patients were included in this study. Based on the predefined MFBIA criteria, 33 patients and 20 patients comprised the clinical lymphedema and subclinical lymphedema groups, respectively. The mean age of the participants was 57.68 ± 8.36 years, and their mean body mass index was 23.73 ± 3.37 kg/m^2^. The surgery side was similarly distributed (right, 49%; left, 51%). Lymphedema was observed on the dominant side in 58% of patients. Regarding lymph node surgery, 17% of patients underwent sentinel lymph node biopsy, 39% underwent axillary lymph node dissection, and 43% underwent both sentinel lymph node biopsy and axillary lymph node dissection. Most patients received chemotherapy (96%) and radiotherapy (92%). No significant differences in baseline clinical characteristics were observed between groups (Table 1). 

### 3.2. Shear Wave Elastography Findings

The overall comparison of limbs indicated that the SWV of the ipsilateral limb was significantly higher than that of the contralateral limb in both the upper arm and forearm (upper arm: *p* = 0.021; forearm: *p* = 0.022). However, the subgroup analysis revealed that these differences were observed only in the clinical lymphedema group.

In the clinical lymphedema group, the SWV of the ipsilateral limb was significantly higher than that of the contralateral limb in both the upper arm and forearm (upper arm: *p* = 0.050; forearm: *p* = 0.048). In contrast, no significant differences in the SWV of the ipsilateral and contralateral limbs were observed in the subclinical lymphedema group at either site (Table 2). Representative SWE images of patients with clinical and subclinical lymphedema are shown in Figure 2.

### 3.3. Tissue Thickness Changes

**Figure 3:**
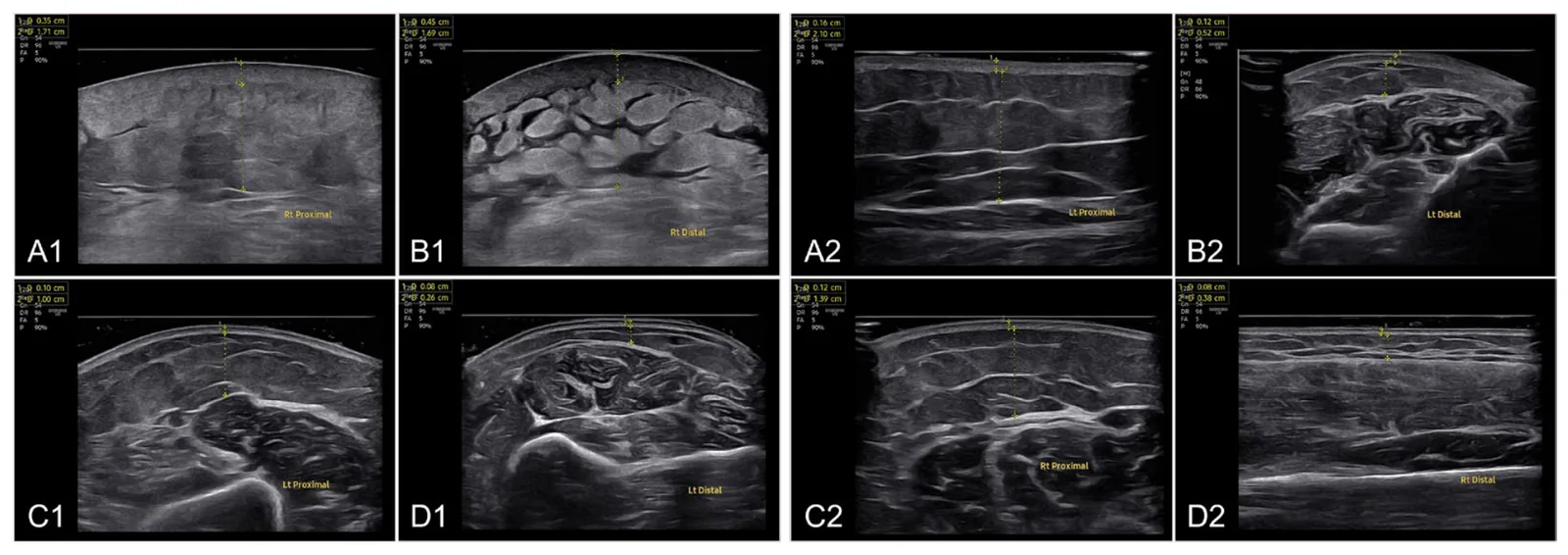
Representative ultrasound images of dermal and subcutaneous thickness measurements of patients with clinical and subclinical lymphedema. (**A1**–**D1**) Images of a representative patient with clinical lymphedema: ipsilateral upper arm (**A1**), ipsilateral forearm (**B1**), contralateral upper arm (**C1**), and contralateral forearm (**D1**). (**A2**–**D2**) Images of a representative patient with subclinical lymphedema: ipsilateral upper arm (**A2**), ipsilateral forearm (**B2**), contralateral upper arm (**C2**), and contralateral forearm (**D2**). The “+” symbols represent the measurement calipers, and the dotted lines connecting the calipers indicate the measured tissue thickness.

An analysis of tissue thickness revealed that the dermal thickness and subcutaneous thickness in the ipsilateral limb were significantly greater than those in the contralateral limb in the overall cohort as well as in the clinical and subclinical lymphedema groups (all *p* < 0.01). Representative ultrasound images of dermal thickness and subcutaneous thickness measurements are shown in Figure 3.

Upper arm dermal thickness in the ipsilateral limb was significantly higher than that in the contralateral limb (0.19 ± 0.05 mm vs. 0.14 ± 0.03 mm; *p* < 0.001), and similar findings were observed in the forearm (0.16 ± 0.06 mm vs. 0.11 ± 0.02 mm; *p* < 0.001). These differences were also observed in the subclinical group (Table 3).

A comparison of the clinical and subclinical lymphedema groups revealed that the dermal thickness ratio (ipsilateral/contralateral) of the upper arm and that of the forearm were significantly higher (upper arm: *p* < 0.001; forearm: *p* = 0.021) in the clinical group. In contrast, the subcutaneous thickness ratio did not significantly differ between groups (Table 4).

### 3.4. Bioelectrical Impedance Analysis Parameters

A comparison of bioelectrical impedance analysis (BIA) parameters revealed that the values across all measured indices, including the ECW ratio, 5 kHz impedance ratio, and phase angle ratio, in the clinical lymphedema group were significantly higher than those in the subclinical group (all *p* < 0.001). The calculation direction of each ratio was selected so that higher values consistently indicated greater lymphedema severity across all bioimpedance parameters. Specifically, the ECW ratio (ipsilateral/contralateral) in the clinical lymphedema group was higher than that in the subclinical lymphedema group. Additionally, both the 5 kHz impedance ratio (contralateral/ipsilateral) and phase angle ratio (contralateral/ipsilateral) in the clinical lymphedema group were significantly higher than those in the subclinical lymphedema group (Table 5).

### 3.5. Correlation Analysis

A correlation analysis of the overall population demonstrated significant positive correlations among BIA parameters. The ECW ratio showed significant positive correlations with both the 5 kHz impedance ratio (ρ = 0.847; *p* < 0.01) and phase angle ratio (ρ = 0.941; *p* < 0.01). Additionally, a significant correlation between the 5 kHz impedance ratio and phase angle ratio was observed (ρ = 0.680; *p* < 0.01).

The BIA parameters were also significantly correlated with tissue thickness measurements. The ECW ratio was significantly correlated with upper arm dermal thickness (ρ = 0.582; *p* < 0.01), forearm dermal thickness (ρ = 0.504; *p* < 0.01), and forearm subcutaneous thickness (ρ = 0.365; *p* < 0.01). Similarly, the 5 kHz impedance ratio showed significant correlations with dermal thickness in the upper arm and forearm (ρ = 0.585 and ρ = 0.555, respectively; both *p* < 0.01), as well as with subcutaneous thickness in the forearm (ρ = 0.333; *p* < 0.01). The phase angle ratio was also significantly correlated with upper arm dermal thickness (ρ = 0.510; *p* < 0.01), forearm dermal thickness (ρ = 0.436; *p* < 0.01), and forearm subcutaneous thickness (ρ = 0.317; *p* < 0.01).

**Figure 4:**
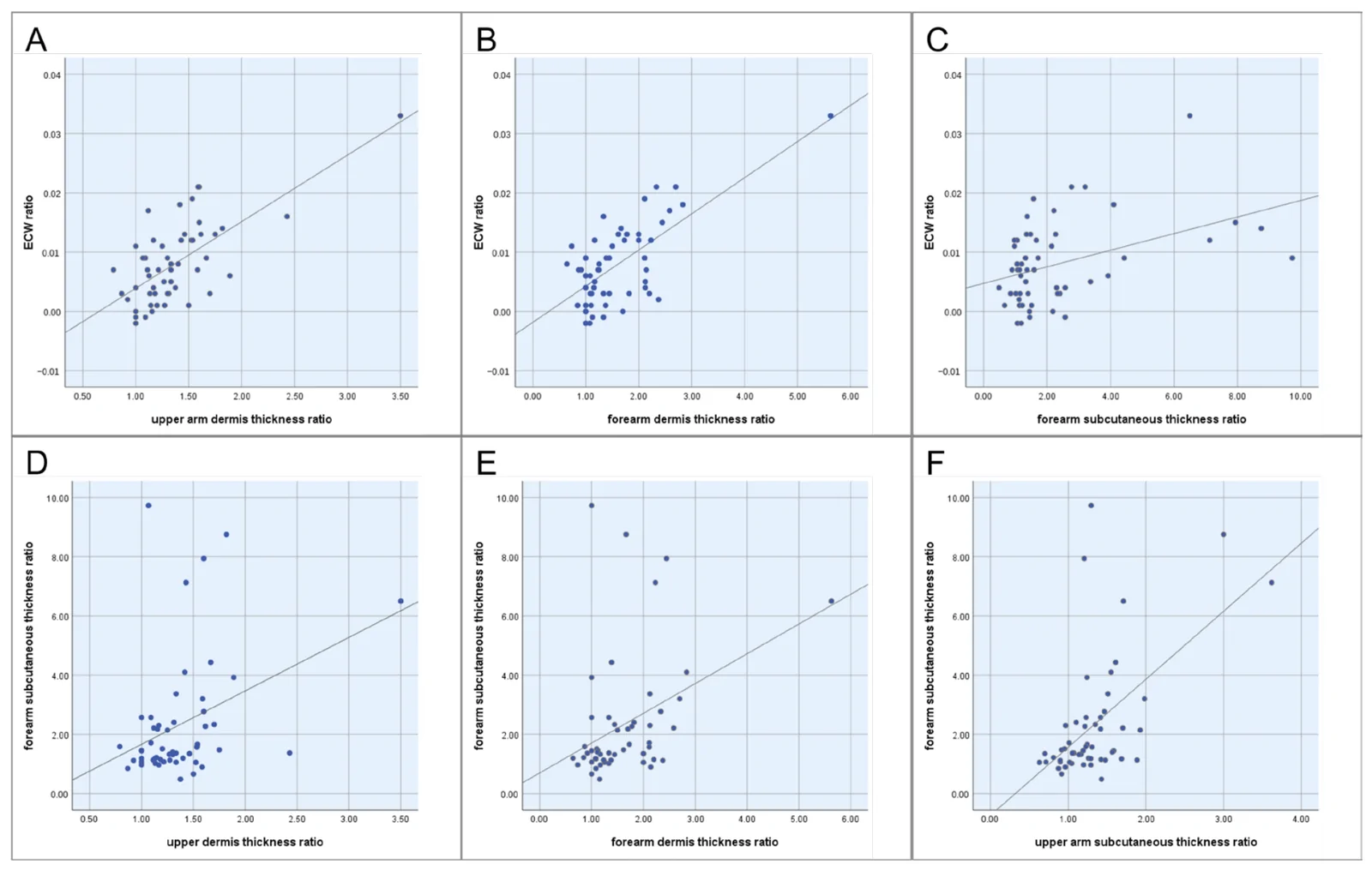
Correlations between tissue thickness ratios and bioimpedance parameters of patients with lymphedema. (**A**) Upper arm dermal thickness ratio versus the ECW ratio. (**B**) Forearm dermal thickness ratio versus the ECW ratio. (**C**) Forearm subcutaneous thickness ratio versus the ECW ratio. (**D**) Upper arm dermal thickness ratio versus the forearm subcutaneous thickness ratio. (**E**) Forearm dermal thickness ratio versus the forearm subcutaneous thickness ratio. (**F**) Upper arm subcutaneous thickness ratio versus the forearm subcutaneous thickness ratio. Each blue dot represents the measurements from an individual participant.

Regarding correlations between tissue thickness parameters, forearm subcutaneous thickness was significantly positively correlated with upper arm dermal thickness (ρ = 0.338; *p* < 0.01), forearm dermal thickness (ρ = 0.390; *p* < 0.01), and upper arm subcutaneous thickness (ρ = 0.491; *p* < 0.01). In contrast, SWE parameters were not significantly correlated with the BIA parameters and tissue thickness measurements, and no significant associations with SWE values in the upper arm and forearm were observed (Table 6, Figure 4).

## 4. Discussion

In this study, we performed an integrated evaluation of BCRL using the MFBIA, ultrasound-based tissue thickness measurements, and SWE to investigate the pathophysiological characteristics reflected by each modality and their stage-dependent differences.

The present study demonstrated three principal findings. First, morphological changes, including increased dermal thickness and subcutaneous thickness, were already evident in the subclinical stage, indicating that these changes represent early manifestations of lymphedema. These findings are consistent with those of previous studies that reported increased dermal thickness and subcutaneous thickness in the ipsilateral limbs of patients with lymphedema, as demonstrated by ultrasound-based assessments [35]. Second, fluid-related parameters derived from the MFBIA demonstrated significant differences between clinical and subclinical lymphedema, thus supporting their utility for differentiating disease stages. This finding is consistent with those of prior studies that reported that the BIA enables detection of early fluid alterations before the onset of clinically apparent lymphedema and facilitates the identification of subclinical disease [26]. Third, increased tissue stiffness measured by SWE was observed only in the clinical lymphedema group, indicating that changes in tissue stiffness predominantly occur during later disease stages. This finding is consistent with those of previous studies that demonstrated that SWE is more sensitive to later-stage lymphedema associated with fibrotic tissue changes rather than early-stage disease. Erdogan Iyigun et al. reported that although elastographic differences were not evident in stage 1 lymphedema, they became significant in stage 2, suggesting that tissue stiffness increases after subcutaneous fibrosis development [36]. Similarly, Lee and Cho reported increased cutaneous stiffness measured by SWE in patients with BCRL, supporting the utility of SWE as a marker of fibrotic tissue remodeling [28].

In this context, our findings further support the notion that SWE reflects a distinct pathophysiological component of lymphedema primarily related to fibrosis and tissue remodeling; therefore, it may provide complementary information for fluid-based and morphology-based assessments across different disease stages.

In the subclinical stage of lymphedema, dermal thickness measured by ultrasound was significantly increased; however, no significant changes in tissue stiffness were detected by SWE. These findings indicate that structural alterations begin early in the disease process, prior to the development of measurable changes in tissue stiffness. In particular, increased dermal thickness may be attributable to the accumulation of lymphatic and interstitial fluid accompanied by early inflammatory responses. Collectively, these findings suggest that the subclinical stage represents an early phase of inflammation and edema in which impaired lymphatic drainage initiates fluid collection within the dermis without yet producing the fibrotic changes necessary to alter tissue stiffness. The absence of changes detected by SWE at this stage implies that the tissue architecture, although morphologically altered, retains near-normal mechanical properties.

Of note, a direct comparison of the clinical and subclinical groups revealed that the dermal thickness ratio was significantly greater in the clinical group; however, the subcutaneous thickness ratio at both sites did not significantly differ between groups. These findings indicate that, compared with subcutaneous thickening, dermal thickening is more closely associated with disease severity.

Furthermore, significant correlations were observed between BIA parameters and dermal thickness, suggesting that dermal thickening is closely associated with and may reflect underlying fluid accumulation in lymphedema.

Previous studies have reported that chronic lymphedema is characterized by progressive fibrotic remodeling and adipose tissue deposition, which gradually replace the initial fluid-dominant phase [4]. In the present study, the absence of significant changes in SWE values in the subclinical stage suggested that these structural alterations have not yet progressed sufficiently to increase tissue stiffness. Therefore, the pathological changes observed in subclinical lymphedema appear to be dominated by fluid accumulation and early tissue remodeling rather than established fibrosis.

In the clinical stage, significant increases in SWE values were observed with elevated MFBIA parameters, suggesting the coexistence of fluid accumulation and increased tissue stiffness. Additionally, the higher phase angle ratio observed in the clinical group may indicate the presence of cellular alterations associated with lymphedema progression. Because the phase angle reflects cellular membrane integrity and tissue composition rather than the fluid volume itself, its elevation suggests that the transition to clinical lymphedema involves tissue-level cellular changes beyond simple extracellular fluid accumulation [30,31,32]. This interpretation is consistent with the established pathophysiology of lymphedema in which persistent protein-rich fluid accumulation promotes fibroblast activation and extracellular matrix deposition, thus leading to progressive tissue remodeling and fibrosis. Accordingly, the increased SWE values observed in the clinical group may reflect progressive tissue remodeling associated with lymphedema. However, lymphedematous tissue is highly heterogeneous and consists of varying proportions of interstitial fluid, fibrosis, adipose tissue, fascia, and postoperative scar tissue, all of which have different mechanical properties. Therefore, SWE measurements should not be interpreted as a fibrosis-specific biomarker, but rather as representing the composite mechanical behavior of the affected tissue.

A major strength and novelty of this study is its multimodal, stage-specific design. Although most prior studies have used a single assessment modality, the present study simultaneously integrated MFBIA, ultrasound, and SWE across clearly defined disease stages. This approach enabled characterization of stage-specific biomarker profiles and, importantly, allowed us to propose a hypothetical stage-associated biomarker pattern. Specifically, ultrasound-based tissue thickness measurements were increased in both the clinical and subclinical groups, MFBIA parameters distinguished the clinical and subclinical stages, and SWE demonstrated significant stiffness changes only in the clinical group. Collectively, our findings suggest that MFBIA, ultrasound, and SWE provide complementary information that may improve stage-specific assessment and longitudinal monitoring of breast cancer-related lymphedema. Given that complete recovery from established BCRL remains challenging, individualized stage-specific assessment using multimodal evaluation may facilitate timely intervention and the development of appropriate treatment strategies.

This study had several limitations. First, it was conducted at a single center and included a relatively small sample size, which may have introduced selection bias and limited the generalizability of results. Moreover, as an exploratory multimodal study involving multiple statistical comparisons without formal correction for multiple testing, findings with borderline significance should be interpreted with caution, and adequately powered future studies incorporating appropriate multiple-comparison correction and multivariable analyses are warranted to confirm these findings. Second, the cross-sectional design precluded an assessment of temporal changes in lymphedema and limited the ability to establish causal relationships among the measured parameters. Therefore, the proposed hypothetical stage-associated biomarker pattern was derived from cross-sectional observations rather than direct evidence of disease progression. This hypothesis requires confirmation through longitudinal studies. Third, ultrasound and SWE measurements are operator-dependent and may be influenced by factors such as ROI placement, probe pressure, and tissue heterogeneity. In particular, the heterogeneous nature of lymphedematous tissue may contribute to variability in stiffness measurements. In addition, this study focused on quantitative ultrasound biomarkers and did not include qualitative gray-scale ultrasound assessments (e.g., tissue architecture, scar tissue, or cobblestone appearance) or Doppler evaluation of vascular alterations, both of which may provide complementary information regarding lymphedema. Furthermore, ultrasound assessments were performed by a single experienced physiatrist at two anatomically standardized sites to improve measurement consistency. Future studies should incorporate whole-limb ultrasound mapping to better account for spatial tissue heterogeneity and formal interobserver reliability analyses to establish measurement reproducibility. Fourth, several potentially important clinical variables, including time since radiotherapy, ISL stage, previous lymphedema treatment, detailed surgical information, and treatment-related neurological complications, were not systematically collected. Furthermore, because a postoperative control group without lymphedema was not included, treatment-related tissue changes could not be completely distinguished from early lymphedema-related changes. These factors should be considered in future studies. Fifth, because there is currently no universally accepted independent reference standard for subclinical BCRL, patient classification in this study was based on the previously validated 5 kHz impedance ratio derived from MFBIA. Therefore, the observed differences between the clinical and subclinical groups should not be interpreted as an independent validation of MFBIA itself. Sixth, this study did not include direct comparisons with reference imaging modalities such as lymphoscintigraphy or indocyanine green lymphography; therefore, the diagnostic accuracy of each modality could not be directly validated. As outlined in the introduction, this reflects the specific aim of the present study to evaluate noninvasive, readily repeatable tools suitable for longitudinal follow-up, given the practical constraints associated with ICG lymphography, including injection-related discomfort and the need for specialized equipment. Future longitudinal studies incorporating direct comparison with ICG lymphography or lymphoscintigraphy would help clarify whether the proposed stage-associated biomarker pattern reflects different phases of disease progression and further validate the diagnostic value of the noninvasive multimodal approach against these reference standards. Current management of BCRL is primarily based on conservative interventions, including compression therapy, manual lymphatic drainage, and remedial exercise. Investigating changes in the MFBIA, ultrasound, and SWE parameters in response to therapeutic interventions may help establish these modalities as biomarkers of the treatment response and support stage-specific treatment monitoring. Furthermore, integrating multimodal data into predictive models or artificial intelligence-based diagnostic systems may improve early detection and risk stratification of lymphedema [37,38,39].

## 5. Conclusions

Our findings suggest that morphological changes, fluid alterations, and tissue stiffness emerge at different time points and exhibit a stepwise pattern throughout the progression of lymphedema. The ultrasound-based assessment of dermal thickness and subcutaneous thickness may be a screening tool for detecting early morphological changes, whereas the MFBIA may be a quantitative modality for evaluating fluid alterations and distinguishing clinical stages of lymphedema. Additionally, SWE may be an adjunctive tool for assessing fibrosis and disease chronicity. These findings support the complementary roles of each assessment modality and underscore the importance of a multimodal approach in the evaluation of lymphedema. Overall, this multimodal approach may facilitate a stage-specific evaluation of lymphedema, thus improving individualized treatment strategies and longitudinal monitoring of disease progression.

## Figures and Tables

**Table 1:** Demographic and Clinical Data of the Participants.

Demographics	Total (*n* = 53)	Subclinical (*n* = 20)	Clinical (*n* = 33)	*p*-Value
Age at diagnosis, y	57.68 ± 8.36	55 ± 7.80	59.3 ± 8.38	0.069
BMI at baseline (kg/m^2^)	23.73 ± 3.37	23.44 ± 3.25	23.9 ± 3.47	0.635
Operation site, n (%)				
Rt/Lt	26 (49)/27 (51)	10 (50)/10 (50)	16 (48)/17 (52)	1.000
Lymphedema side same as dominant hand, n (%)	31 (58)	11 (55)	20 (61)	0.772
Type of Lymph node surgery, n (%)				0.126
SLNB	9 (17)	6 (30)	3 (9)	
ALND	21 (39)	6 (30)	15 (45.5)	
ALND c SLNB	23 (43)	8 (40)	15 (45.5)	
Chemotherapy, n (%)	51 (96)	19 (95)	32 (97)	1.000
Radiation therapy, n (%)	49 (92)	19 (95)	30 (91)	1.000
Time since surgery, months	76.08 ± 60.71	75.99 ± 48.91	76.15 ± 67.59	0.516
Comorbidities, n (%)				
Hypertension	13 (25)	4 (20)	9 (27)	0.744
Diabetes mellitus	6 (11)	1 (5)	5 (15)	0.390
Hyperlipidemia	14 (26)	4 (20)	10 (30)	0.527

Values are presented as mean ± SD or as number (%). *p*-values were obtained using the independent *t*-test (age, BMI), the Mann–Whitney U test (time since surgery), and Fisher’s exact test for categorical variables. Abbreviations: SLNB, Sentinel lymph node biopsy; ALND, axillary lymph node dissection.

**Table 2:** Comparison of Shear Wave Elastography Values Between Ipsilateral and Contralateral Limbs.

Group	Site	Ipsilateral (m/s)	Contralateral (m/s)	*p*-Value	Effect Side (r)
Total (*n* = 53)	Upper arm	4.73 ± 1.74	4.06 ± 1.47	0.021 *	0.35
	Forearm	4.8 ± 1.19	4.35 ± 1.3	0.022 *	0.32
Subclinical (*n* = 20)	Upper arm	4.66 ± 1.84	3.95 ± 1.53	0.108	0.36
	Forearm	4.99 ± 1.16	4.58 ± 1.42	0.305	0.23
Clinical (*n* = 33)	Upper arm	4.77 ± 1.7	4.12 ± 1.45	0.050 *	0.34
	Forearm	4.7 ± 1.2	4.22 ± 1.22	0.048 *	0.34

Data are presented as mean ± SD. r, effect size for the Wilcoxon signed-rank test. * *p* < 0.05; Wilcoxon signed-rank test.

**Table 3:** Comparison of Tissue Thickness between Ipsilateral and Contralateral Limbs.

Parameters	Total (*n* = 53)			Subclinical (*n* = 20)			Clinical (*n* = 33)		
	Ipsilateral	Contralateral	*p* Value	Ipsilateral	Contralateral	*p* Value	Ipsilateral	Contralateral	*p* Value
Dermis thickness, mm									
Upper arm	0.19 ± 0.05	0.14 ± 0.03	<0.001 *	0.16 ± 0.03	0.14 ± 0.03	0.006 *	0.2 ± 0.06	0.14 ± 0.03	<0.001 *
Forearm	0.16 ± 0.06	0.11 ± 0.02	<0.001 *	0.14 ± 0.03	0.11 ± 0.03	0.005 *	0.18 ± 0.07	0.11 ± 0.02	<0.001 *
Subcutaneous tissue thickness, mm									
Upper arm	1.37 ± 0.55	1.10 ± 0.41	<0.001 *	1.32 ± 0.65	1.09 ± 0.46	0.002 *	1.4 ± 0.49	1.1 ± 0.38	0.001 *
Forearm	0.7 ± 0.33	0.42 ± 0.22	<0.001 *	0.63 ± 0.37	0.4 ± 0.2	<0.001 *	0.75 ± 0.31	0.44 ± 0.24	<0.001 *

Data are presented as mean ± SD. * *p* < 0.05; Wilcoxon signed-rank test.

**Table 4:** Comparison of Tissue Thickness Ratios between Clinical and Subclinical Stage of Lymphedema.

Stage	Dermis Thickness		Subcutaneous Tissue Thickness	
	Upper Arm Ratio (Ips./Cont.)	Forearm Ratio (Ips./Cont.)	Upper Arm Ratio (Ips./Cont.)	Forearm Ratio (Ips./Cont.)
Clinical	1.494 ± 0.478	1.782 ± 0.906	1.391 ± 0.609	2.713 ± 2.511
Subclinical	1.142 ± 0.144	1.319 ± 0.435	1.222 ± 0.248	1.661 ± 0.665
*p*-value	<0.001 **	0.021 *	0.545	0.569
Effect size (r)	0.49	0.32	0.08	0.08

Ips./Cont. = ipsilateral limb/contralateral limb. Data are presented as mean ± SD. r, effect size for the Mann–Whitney U test. * *p* < 0.05, ** *p* < 0.01; Mann–Whitney U test.

**Table 5:** Comparison of Bioelectrical Impedance Analysis Parameters between Clinical and Subclinical Lymphedema Patients.

Parameters	Subclinical (*n* = 20)	Clinical (*n* = 33)	*p*-Value	Effect Size (r)
ECW ratio (ipsilateral arm/contralateral arm)	0.003 ± 0.003	0.011 ± 0.007	<0.001 *	0.66
5 kHz Impedance ratio (contralateral arm/ipsilateral arm)	1.040 ± 0.032	1.302 ± 0.193	<0.001 *	0.80
Phase angle ratio (contralateral arm/ipsilateral arm)	1.086 ± 0.125	1.233 ± 0.192	<0.001 *	0.50

Data are presented as mean ± SD. r, effect size for the Mann–Whitney U test. * *p* < 0.05; Mann–Whitney U test.

**Table 6:** Correlations between BIA Parameters, Tissue Thickness Ratio and Shear-Wave Elastography Ratio in Total Patients.

Parameters	BMI	ECW R	5 kHz IR	PhA R	U-Derm R	F-Derm R	U-Subcu R	F-Subcu R	U-SWE R	F-SWE R
BMI	1.000	0.185 (−0.09–0.43)	0.008 (−0.26–0.28)	0.171 (−0.10–0.42)	−0.019 (−0.29–0.25)	0.017 (−0.25–0.29)	0.130 (−0.15–0.39)	−0.180 (−0.43–0.09)	0.167 (−0.11–0.42)	0.038 (−0.23–0.31)
ECW R		1.000	0.847 ** (0.75–0.91)	0.941 ** (0.90–0.97)	0.582 ** (0.37–0.74)	0.504 ** (0.27–0.68)	0.250 (−0.02–0.49)	0.365 ** (0.11–0.58)	0.137 (−0.14–0.39)	0.195 (−0.08–0.44)
5 kHz IR			1.000	0.680 ** (0.50–0.80)	0.585 ** (0.37–0.74)	0.555 ** (0.33–0.72)	0.178 (−0.10–0.43)	0.333 ** (0.07–0.55)	−0.018 (−0.29–0.43)	0.153 (−0.12–0.41)
PhA R				1.000	0.510 ** (0.28–0.69)	0.436 ** (0.19–0.63)	0.184 (−0.09–0.43)	0.317 ** (0.05–0.54)	0.183 (−0.09–0.43)	0.202 (−0.07–0.45)
U-derm R					1.000	0.427 ** (0.18–0.63)	0.157 (−0.12–0.41)	0.338 ** (0.07–0.56)	0.170 (−0.11–0.42)	0.125 (−0.15–0.38)
F-derm R						1.000	0.228 (−0.05–0.47)	0.390 ** (0.13–0.60)	0.063 (−0.21–0.33)	0.269 (0.00–0.50)
U-subcu R							1.000	0.491 ** (0.25–0.67)	0.158 (−0.12–0.41)	−0.199 (−0.45–0.08)
F-subcu R								1.000	0.043 (−0.23–0.31)	0.015 (−0.26–0.28)
U-SWE R									1.000	0.197 (−0.08–0.44)
F-SWE R										1.000

Values are presented as Spearman’s rho, with 95% confidence intervals shown in parentheses. IR = impedance ratio; PhA R = phase angle ratio; ECW R = extracellular water ratio; U = upper arm; F = forearm. Impedance and phase angle ratios were defined as contralateral/ipsilateral arm, whereas ECW ratio was defined as ipsilateral/contralateral arm. ** *p* < 0.01.

## Data Availability

The datasets generated and/or analyzed during the current study are available from the corresponding author on reasonable request.

## Abbreviations

The following abbreviations are used in this manuscript:

BCRL	Breast cancer-related lymphedema
BIA	Bioelectrical impedance analysis
ECW	Extracellular water
MFBIA	Multiple-frequency bioelectrical impedance analysis
ROI	Region of interest
SWE	Shear wave elastography
SWV	Shear wave velocity
